# SHORT- AND LONG-TERM OUTCOMES AFTER HIP SURGERY IN OLDER ADULTS WITH AND WITHOUT HEART FAILURE

**DOI:** 10.1093/geroni/igac059.3052

**Published:** 2022-12-20

**Authors:** Sijia Wei, Wei Pan, Tingzhong (Michelle) Xue, Hideyo Tsumura, Chiyoung Lee, Eleanor McConnell

**Affiliations:** Northwestern University Feinberg School of Medicine, Chicago, Illinois, United States; Duke University, Durham, North Carolina, United States; Duke University School of Nursing, Durham, North Carolina, United States; University of Michigan School of Nursing, Durham, North Carolina, United States; University of Washington Bothell, Bothell, Washington, United States; Duke University, Durham, North Carolina, United States

## Abstract

Older adults with heart failure (HF) have a higher risk for adverse outcomes after hip fracture surgery than those without. Propensity score matching (PSM) reduces selection bias and makes a direct group comparison (older adults with and without HF) possible. Thus, this study aimed to assess the impact of HF on short-and long-term outcomes after hip fracture surgery in older adults living with and without HF. Electronic health records data of older adults (n = 1171) hospitalized for hip fracture surgery between October 2015 and December 2018 were extracted. Comparison groups (with and without HF) were identified using 1:1 ratio PSM to control for observed differences in baseline characteristics. Regression models were used to compare group differences in outcomes. Although in analyses without PSM, older adults with HF were more likely to have higher 90-day readmission, and 30-, 90-, and 365-day mortality, this association was not significant after controlling for selection bias. However, the associations between having HF with 30-day readmission and longer length of stay were significant before and after PSM. Additionally, if patients did not receive hip fracture surgery procedures within two days of admission, they had a 3.6-day longer inpatient stay (P-value < 0.0001) and were 47.8 times more likely to die during hospitalization (95%CI 4.9–482.0, P-value < 0.001). Being non-white was significantly associated with higher 90- and 365-day mortality. Future research should consider PSM approach on national representative datasets to rigorously evaluate the effects of HF on mortality and readmission following hip fracture surgery in older adults.

